# UC-II Undenatured Type II Collagen for Knee Joint Flexibility: A Multicenter, Randomized, Double-Blind, Placebo-Controlled Clinical Study

**DOI:** 10.1089/jicm.2021.0365

**Published:** 2022-06-07

**Authors:** Christiane Schön, Katharina Knaub, Wilfried Alt, Shane Durkee, Zainulabedin Saiyed, Vijaya Juturu

**Affiliations:** ^1^ Nutritional CRO, BioTeSys GmbH, Esslingen, Germany.; ^2^ Institute of Sports Science and Kinesiology, University of Stuttgart, Stuttgart, Germany.; ^3^ Research and Development, Lonza Greenwood, North Emerald Road, Greenwood, SC, USA.

**Keywords:** undenatured collagen, joint flexibility, range of motion, knee joint function

## Abstract

**Objective:**

Joint-related stress models have been used in the past to induce a standardized load on physical structures, allowing researchers to observe changes in perceived stress on joints as accurately as possible in healthy individuals. Previous studies support the efficacy of UC-II^®^ undenatured type II collagen (“undenatured collagen”) supplementation in maintaining joint health. The purpose of this study was to assess the effect of undenatured collagen on knee flexibility in healthy subjects who experience activity-related joint discomfort (ArJD).

**Methods:**

This randomized, double-blind, placebo (PLA)-controlled study was conducted in healthy subjects with ArJD who had no history of osteoarthritis, or joint diseases. Ninety-six (*n* = 96, 20–55 years old) subjects who reported joint discomfort while performing a standardized single-leg-step-down test were randomized to receive either PLA (*n* = 48) or 40 mg of undenatured collagen (*n* = 48) supplementation daily for 24 weeks. Range of motion (ROM) flexion and extension were measured using a digital goniometer.

**Results:**

At the end of the study, a statistically significant increase in knee ROM flexion was observed in the undenatured collagen group versus the PLA group (3.23° vs. 0.21°; *p* = 0.025). In addition, an increase in knee ROM extension by 2.21° was observed over time in the undenatured collagen group (*p* = 0.0061), while the PLA group showed a nonsignificant increase by 1.27° (*p* > 0.05). Subgroup analysis by age showed a significant increase in knee ROM flexion in subjects >35 years old in the undenatured collagen supplemented group compared with PLA (6.79° vs. 0.30°; *p* = 0.0092).

**Conclusion:**

Overall, these results suggest that daily supplementation of 40 mg of undenatured collagen improved knee joint ROM flexibility and extensibility in healthy subjects with ArJD.

## Introduction

Arthritis is a chronic degenerative joint disease that impacts the mobility and physical functioning in affected individuals.^1^ Osteoarthritis (OA) is the most common form of arthritis that involves destruction of joint cartilage and damage to the adjacent bone. Furthermore, OA is the most prevalent joint disease in the United States and its prevalence has approximately doubled since the mid-20th century.^2^ According to the Centers for Disease Control (CDC), in 2020, an estimated 32.2 million Americans were living with OA.^1,3^ As common treatment of OA symptoms, prescription of oral nonsteroidal anti-inflammatory drugs (NSAIDs) is widely used. In a recent individual patient data meta-analysis, Persson et al. showed that topical NSAIDs are effective for OA pain relief.^4^

Daily life activity is characterized by differing intensities of physical activity that exert variable weight-bearing load on the joints. Joint stress caused by mechanical overload, anatomical weaknesses (e.g., unequal leg length and knock knees) or joint instability leads to localized pain and stiffness that limit joint flexibility and mobility in healthy subjects without diagnosed OA.^5^ Studies have shown that even a few degrees of loss of knee range of motion (ROM) flexibility can result in altered gait patterns leading to difficulty in running and jumping.^6,7^ Knee ROM is essential for daily function for athletes as well as for normal active people.^8^ Interventions aimed at improving ROM have been shown to alleviate joint stiffness, increase joint mobility, and maintain joint function.

A recent investigation of Wallace et al. on long-term trends in knee OA prevalence in the United States indicated that knee OA may be more preventable than is currently supposed.^2^ Therefore, preventive actions, which include joint protection by physical activity, dietary intervention, or dietary supplements, appear to be an important factor in the progression of this disease. Undenatured collagen is one such dietary supplement that could be used in subjects with activity-related joint discomfort (ArJD) to prevent possible progression of the complaints such as limited mobility. Studies have shown that undenatured collagen supplementation can improve joint mobility in OA subjects as well as in healthy subjects who experience ArJD.^9,10^

In a placebo-controlled study, Lugo et al. reported improvements in knee joint extension in healthy subjects supplemented with undenatured collagen and who experienced joint pain while performing the stepmill exercise.^10^ More recently, our group validated the single-leg-step-down (SLSD) test as a reliable model to select for healthy subjects who experience ArJD, thereby allowing assessment of knee joint function in this population.^11^ In this study, Schön et al. demonstrated that subjects with ArJD may show impairments of knee joint flexibility assessed by goniometry in comparison with the healthy subjects without any joint complaints. Therefore, the purpose of the current study was to evaluate the impact of undenatured collagen supplementation on joint flexibility, as measured by knee ROM flexion and extension in healthy subjects who experience ArJD on the SLSD test.

## Materials and Methods

### Study design

The study was performed as a prospective, multicentric, randomized, double-blind, placebo-controlled study in parallel design. This study was conducted following the guidelines for Good Clinical Practice set forth by the International Council for Harmonization of Technical Requirements for Pharmaceuticals for Human Use (ICH E6 [R2], Nov.2016) and following the Declaration of Helsinki (E8) for treatment of human subjects in a study. This study was approved by the local ethics committee (Institutional Review Board of the Landesärztekammer Baden-Württemberg, file number F-2019-072) and the clinical trial was registered at DRKS—German Clinical Trials Register DRKS: DRKS00018792.

Subjects were screened for their eligibility after providing written informed consent. All subjects completed a medical history questionnaire at screening. Subjects were assessed for anthropometric measures and vital signs. Healthy males and females, 20–55 years old with a body mass index between 19 and 29.9 kg/m^2^, were eligible to participate in the study.

All subjects had to perform sports at least two times per week, including but not limited to activities such as soccer, basketball, handball, volleyball, tennis, and running. In addition, subjects had to report reversible knee-joint discomfort during or immediately after physical activity over a period of at least 3 months. The SLSD test was further used to select subjects at the screening visit.^11,12^ Only subjects who experienced a pain level of five on an 11-point Likert scale while performing between 30 and 150 steps during the SLSD test were eligible to participate.

Exclusion criteria included joint replacement of the knee, planned surgical intervention during the study duration, intra-articular therapy within the 3 months before the study initiation, and a history or presence of any medical disorders that could potentially interfere with the study, such as active cancer, cardiovascular disease (e.g., stroke and heart attack), or pregnancy and lactation. In addition, subjects with hip, spine, or foot injuries were excluded. Further exclusion criteria were smoking of more than five cigarettes per day, known hypersensitivity to eggs, chicken, or any ingredients in the products, and chronic use of pain relief medication within 30 days before the screening visits.

To reduce the effect of confounding factors, study subjects were asked to maintain their usual diet during the study duration. The use of dietary supplements that could influence joint pain, discomfort, and recovery was not allowed throughout the study. Forty-eight hours before the test days, subjects were not allowed to perform any sporting activities, such as cycling, running, or other exhaustive physical activities, such as heavy gardening or hiking. Thirty-six hours before screening and at all study visits, subjects were asked not to take any oral pain medication (e.g., aspirin and paracetamol) to avoid any possible impact of anti-inflammatory ingredients on joint discomfort or joint flexion. Subjects had to document any intake of pain medication in a diary. In addition, at each study visit, the recent intake of pain relievers was assessed.

**FIG. 1.**
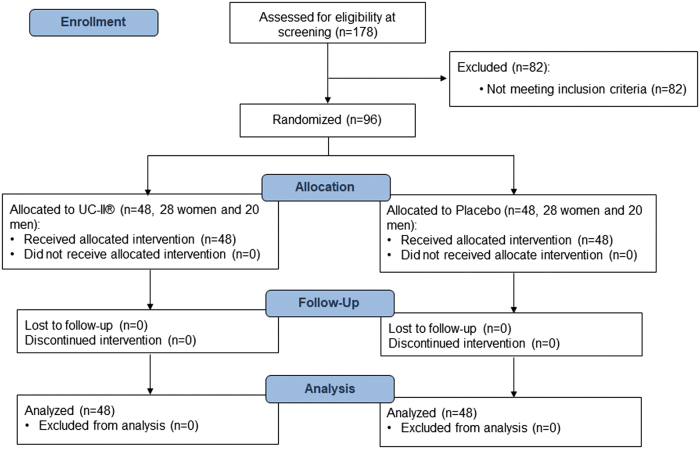
CONSORT flow diagram.

Among the 178 males and females screened, 96 subjects were randomized, and 82 subjects were identified as screen failures according to the inclusion or/and exclusion criteria (Fig. 1). The most common reasons for screen failure were too early occurrence of pain level 5 during the SLSD test (<30 repetitions) or too low pain level during the SLSD test performance (<5). There were no dropouts during the course of the study. The study was conducted at BioTeSys GmbH and at the Institute of Sport and Movement Science of the University of Stuttgart from September 2019 to January 2021.

The study duration was 24 weeks (6 months) with a total of seven study visits in 4-week intervals that included screening, baseline (visit one), 4 weeks (visit two), 8 weeks (visit three), 12 weeks (visit four), 16 weeks (visit five), 20 weeks (visit six), and 24 weeks (visit seven).

Knee ROM flexion was performed at each study visit using a digital goniometer. Knee ROM extension was performed at baseline, and at week 12 and 24. The assessment of study parameters was done on the leg that typically experienced more intense pain after physical activity (target knee).

### Sample size and randomization

Sample size calculations were based on the results of a previously performed pilot study^11^ and a study by Lugo et al.^10^ Assuming an effect size of 0.636, a sample size of 40 subjects per group would provide ∼80% power using a significance level of 5%. Considering a dropout rate of 15%, the study was performed with 48 subjects per group. When subjects fulfilled all the inclusion criteria and none of the exclusion criteria, effectively establishing study eligibility, they were allocated randomly to one of the two study groups according to the randomization list, using consecutive counting following the schedule of their inclusion visit. The randomization was stratified by gender (male and female) in blocks of *n* = 4. The randomization was performed with RandList (Datinf GmbH, Tübingen, Germany).

### Investigational products

Undenatured collagen from chicken sternum (40 mg UC-II^®^ undenatured type II collagen per day, providing ≥3% undenatured type II collagen) and PLA (containing only excipient, microcrystalline cellulose) in sensory identical capsules were supplied by Lonza Greenwood LLC. (Greenwood, SC, USA). Subjects were instructed to consume products as one capsule daily with water in the evening before bedtime. Subjects were asked to document their intake time in a diary. All investigational products were carefully stored at room temperature and in dry conditions until distributed to subjects. At visits one through six, new bottles were handed out with enough study products to last until their next visit. Compliance was calculated based on the dispensed and returned study products.

### SLSD test

The SLSD test is a unilaterally performed test, which was validated in a previous study.^11^ During the test, subjects had to step forward and down from a platform with adjustable height. The down limb brushed the floor with the heel and then returned back up to the platform to full knee extension. The frequency of repetitions was given by a metronome. Subjects were instructed to indicate the pain level of five on NRS-11, where 0 meant “no pain” and a pain of 10 meant “worst pain possible.” After that, the test was stopped. The number of total repetitions was documented. If no joint pain occurred after latest of at least 10 min, the stress test was stopped.

### Knee ROM

The knee ROM of a joint is typically measured by the number of degrees from the starting position of a segment to its position at the end of its full range of movement. In this study, a digital goniometer (Halo Medical Devices, Sydney, Australia) was used to measure knee ROM flexion and extension.

For knee ROM flexion measurement, the axis of a goniometer was placed at the intersection of the thigh and shank at the knee joint. Subjects lay in a prone position with shanks (lower legs) hanging free over the edge of the examination table (position 1). Position 2 was the maximal flexion of the knee joint (Supplementary Fig. S1). During the measurement of active ROM flexion, the position 2 was reached actively by the subject. While for passive ROM flexion, the position 2 was reached using slight pressure by the investigator. Knee ROM extension was measured at baseline, and at week 12 and 24. The subject sat on an examination table with shanks (lower leg) hanging vertical to the floor (position 1), and the ROM from this position to the maximal extension (position 2) was measured (Supplementary Fig. S1).

### Safety

Hematology, liver enzymes, lipid profile, hsCRP, HbA1c, and kidney function parameters were assessed at screening as well as at the final visit at Synlab Medizinisches Versorgungszentrum Leinfelden-Echterdingen, Germany. Blood pressure and heart rate were evaluated at screening, and at week 12 and 24 after 5 min of rest in a sitting position. Adverse events (AEs) were documented during the study duration. Subjective rating of tolerability was assessed at week 24 using a questionnaire rating as “well-tolerated,” “slightly unpleasant,” and “very unpleasant.” The focus of the tolerability assessment was on any gastrointestinal events possibly linked to the intake of the study product as well as the intake regimen and size of capsule.

### Statistical methods

The analysis was performed on an intent-to-treat population. Analysis of covariance (ANCOVA) with baseline value as a covariate was used to analyze the statistical differences between the groups. Changes over time within the study group were evaluated using analysis of variance (ANOVA) repeated measurements or Friedman test as appropriate. *Post hoc* analysis for comparison between baseline and each study visit was performed applying Dunnett's multiple comparison test or Dunn's multiple comparison test as appropriate. A significance level of *p* < 0.05 was used. The analysis was performed with IBM SPSS statistics 24 statistical software (Armonk, NY).

## Results

### Demographics and baseline characteristics

Table 1 presents the baseline characteristics of the study participants. Ninety-six subjects met the eligibility criteria and were randomized to the PLA (*n* = 48) or to the undenatured collagen (*n* = 48) group. No significant differences were observed for baseline characteristics in both the groups (*p* > 0.05).

**Table 1.** Baseline Characteristics and Safety Parameters

Details	Undenatured collagen (*n* = 48)		PLA (*n* = 48)	
Age, year	34.5 ± 1.5		37.8 ± 1.6	
BMI, kg/m^2^	23.91 ± 0.43		24.29 ± 0.38	
Gender, M/F	20/28		20/28	
Frequency of regular sporting activity				
1 × /week	0%		0%	
2 × /week	31%		21%	
3 × /week	35%		44%	
>3 × /week	33%		35%	
Frequency of intensity of physical activity				
Low	—		2%	
Moderate	21%		21%	
High	79%		77%	

Safety parameters				
Parameter	Undenatured collagen Baseline (*n* = 48)	Undenatured collagen End of intervention (*n* = 48)	PLA Baseline (*n* = 48)	PLA End of intervention (*n* = 48)
SBP, mm Hg	126.5 ± 13.6	125.4 ± 13.9	124.8 ± 12.1	120.5 ± 11.5
DBP, mm Hg	77.1 ± 7.8	78.3 ± 9.2	77.7 ± 9	76.5 ± 9.5
Cholesterol, mg/dL				
Men	175.5 ± 24	191.9 ± 23.35	176.5 ± 37.1	186.9 ± 36.08
Women	178.6 ± 34.5	184.6 ± 32.78	186.5 ± 35.2	187.5 ± 28.37
hsCRP, mg/dL				
Men	0.9 ± 0.7	0.7 ± 0.9	1.1 ± 0.8	1.2 ± 2.0
Women	1.7 ± 1.9	2.0 ± 2.5	1.1 ± 1.3	1.1 ± 1.3

BMI, body mass index; DBP, diastolic blood pressure; PLA, placebo; SBP, systolic blood pressure.

### Product compliance

Overall, intake compliance of study products was more than 99% for both the groups (*p* > 0.05).

### Range of motion

**FIG. 2.**
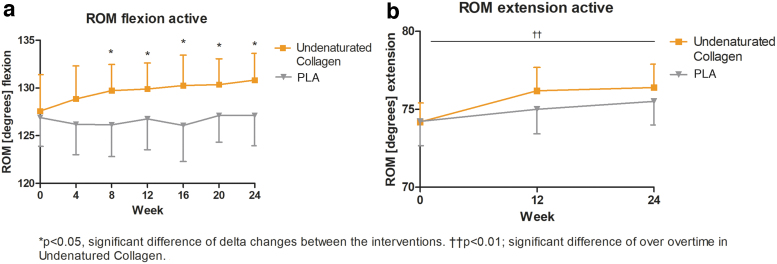
Distribution of ROM results for **(a)** ROM flexion active (°); **(b)** ROM extension active (°) in the undenatured collagen group versus PLA group over the study; *line* graph with mean ± 95% CI; *p < 0.05, significant difference of delta changes between the interventions. ^††^*p* < 0.01; significant difference over time in undenatured collagen. CI, confidence interval; ROM, range of motion; PLA, placebo.

Regarding the parameter ROM flexion active, no significant differences between the study groups were observed at baseline (*p* > 0.05). At the end of the study, the undenatured collagen supplemented group showed a statistically significant mean increase of 3.23° in the ROM flexion compared with the mean increase of 0.21 in PLA° group (undenatured collagen, *n* = 48: 95% confidence interval [CI]: 0.44– 6.02 vs. PLA, *n* = 48: 95% CI: −1.67 to 2.08; *p* = 0.0250). The significant difference between the undenatured collagen group and the PLA group in the ROM flexion active was observed as early as week 8 of supplementation and continued to improve significantly during the course of the study (Fig. 2a). The delta changes from baseline to 24 weeks between the groups are additionally summarized in Table 2 and Supplementary Figure S2.

**Table 2.** Assessment of Range of Motion Parameters at Each Study Visit

Assessment of ROM	Baseline	Week 4	Week 8	Week 12	Week 16	Week 20	Week 24	Delta change after 24 weeks	*p*-Value between*^a^* groups	*p*-Value within*^b^* groups
ROM flexion active (°)										
UC-II	127.6 (123.8–131.4)	128.9 (125.4–132.3)	129.7 (127.0–132.4)	129.9 (127.2–132.6)	130.3 (127.1–133.4)	130.4 (127.6–133.10)	130.8 (128.0–133.6)	3.23 (0.44 to 6.02)	0.025	0.2844
PLA	126.9 (123.9–129.9)	126.2 (123.0–129.4)	126.1 (122.8–129.4)	126.8 (123.5–130.0)	126.1 (122.3–129.8)	127.1 (124.3–129.9)	127.1 (123.9–130.3)	0.21 (−1.67 to 2.08)		0.9134
ROM flexion active (°) by gender										
UC-II female	128.4 (123.1–133.7)	130.5 (126.1–134.9)	131.4 (127.7–135.1)	132.1 (128.9–135.4)	131.8 (128.1–135.5)	131.8 (128.8–134.9)	133.2 (129.7–136.7)	4.79 (1.21 to 8.36)	0.0063	0.0332
PLA female	128 (123.5–132.5)	126.8 (122.5–131.0)	127.1 (122.3–131.9)	128.0 (123.1–133.0)	127.6 (122.2–133.1)	127.9 (123.6–132.2)	127.7 (122.9–132.6)	−0.32 (−2.85 to 2.21)		0.9732
UC-II male	126.4 (120.6–132.2)	126.6 (120.7–132.4)	127.5 (123.3–131.6)	126.8 (122.1–131.4)	128.1 (122.1–1340.0)	128.3 (123.2–133.4)	127.5 (122.9–132.0)	1.05 (−3.60 to 5.70)	0.7881	0.9465
PLA male	125.3 (121.3–129.3)	125.4 (120.2–130.6)	124.8 (120.1–129.5)	125.0 (121.1–129.5)	123.9 (118.6–129.2)	126.0 (122.8–129.1)	126.3 (122.4–130.1)	0.95 (−2.07 to 3.97)		0.8828
ROM flexion active (°) by age										
UC-II age 20–35	130.9 (127.0–134.9)	130.8 (127.0–134.5)	130 (126.9–133.1)	131.2 (128.5–134.0)	133.1 (130.1–136.2)	131.7 (129.0–134.3)	131.8 (129.1–134.5)	0.90 (−2.64 to 4.44)	0.6012	0.3129
PLA age 20–35	130.7 (127.3–134.0)	128.2 (122.8–133.6)	127.0 (120.5–133.4)	129.9 (125.1–134.7)	128.7 (122.4–135.0)	131.0 (127.4–134.7)	130.8 (127.1–134.4)	0.10 (−2.32 to 2.51)		0.2337
UC-II age >35	122.5 (115.1–129.8)	125.9 (119.1–132.8)	129.3 (123.9–134.7)	127.8 (122.2–133.5)	125.8 (119.1–132.3)	128.4 (122.5–134.2)	129.3 (123.2–135.4)	6.79 (2.38 to 11.20)	0.0092	0.0024
PLA age >35	124 (119.4–128.5)	124.6 (120.6–128.6)	125.5 (121.9–129.1)	124.3 (119.9–128.7)	124.0 (119.2–128.8)	124.0 (120.2–127.9)	124.3 (119.5–129.0)	0.30 (−2.60 to 3.19)		0.9462
ROM flexion passive (°)										
UC-II	146.2 (142.9–149.5)	145.7 (142.1–149.3)	148.0 (144.6–151.4)	146.5 (143.1–149.8)	147.9 (143.8–152.0)	147.7 (144.5–150.9)	148.3 (144.4–152.3)	2.15 (−0.71 to 5.00)	0.3103	0.3167
PLA	146.4 (142.9–149.9)	144.3 (140.3–148.2)	144.8 (140.8–148.8)	145.9 (142.1–149.8)	145.6 (141.0–149.8)	145.2 (141.0–150.2)	146.4 (141.9–151.0)	0.06 (−2.85 to 3.97)		0.7695
ROM extension active (°)										
UC-II	74.2 (73.0–75.4)	n.a.	n.a.	76.2 (74.7–77.7)	n.a.	n.a.	76.4 (74.9–77.9)	2.21 (0.82 to 3.60)	0.3115	0.0061
PLA	74.2 (72.7–75.8)	n.a.	n.a.	75.0 (73.4–76.6)	n.a.	n.a.	75.5 (74.0–77.0)	1.27 (−0.17 to 2.71)		0.2540

Values are means (upper and lower bound of 95% CI).

*n* = 48 in each intervention group.

^a^ *p*-Value for comparison between the groups by ANCOVA.

^b^ *p*-Value for within-group analysis by repeated-measures ANOVA or Freidman test as appropriate.

ANCOVA, analysis of covariance; ANOVA, analysis of variance; CI, confidence interval; n.a., not applicable; PLA, placebo; ROM, range of motion; UC-II, Undenaturated type II collagen.

### ROM flexion active according to subgroup gender

Subgroup analysis was performed for knee ROM flexion active based on gender. In the subgroup of females, a statistically significant increase of 4.79° was observed in the undenatured collagen group versus a slight decrease of −0.32° seen in the PLA group (undenatured collagen, *n* = 28: 95% CI: 1.21–8.36 vs. PLA, *n* = 28: 95% CI: −2.85 to 2.21; *p* = 0.0063) after 24 weeks of supplementation (Table 2 and Supplementary Fig. S3). The undenatured collagen group showed a significant increase in ability to flex the knee over baseline (*p* < 0.01) and over time (*p* = 0.0332) among the female subjects, and no such change was observed in the PLA group (*p* > 0.05).

In the subgroup of men, there was a slight nonsignificant increase seen in the ROM flexion for both the groups at the end of the study (undenatured collagen, *n* = 20: +1.05 vs. PLA, *n* = 20: +0.95; *p* > 0.05). The results of the analysis are summarized in Table 2.

### ROM flexion active according to subgroup age

Subgroup analysis was performed for knee ROM flexion active based on age. In the subgroup of age >35 years, subjects in the undenatured collagen group showed a significant increase in ROM flexion than in the PLA group (undenatured collagen, *n* = 19: +6.79°, 95% CI: 2.38–11.20 vs. PLA, *n* = 27: +0.30°, 95% CI: −2.60 to 3.19; *p* = 0.0092). In addition, the undenatured collagen group showed a significant increase in the ability to flex the knee within the group over time (*p* = 0.0024) and over baseline (*p* < 0.01) in subjects >35 years old, while no such change was observed in the PLA group (*p* > 0.05, Table 2).

The results of the group of age >35 years are further summarized in Supplementary Figure S4. In the 20–35-year-old subgroup, there was a slight nonsignificant increase in the ROM at the end of the study in both the groups (undenatured collagen, *n* = 29: 95% CI: −2.64 to 4.44 vs. PLA, *n* = 21: 95% CI: −2.32 to 2.51; *p* > 0.05) (Table 2).

### Knee flexion ROM passive

No significant difference between the study groups was observed at baseline (*p* > 0.05, Table 2). A nonsignificant increase was observed in the undenatured collagen group, while no changes were seen in the PLA group after 24 weeks of intervention (undenatured collagen, *n* = 48: +2.15°, 95% CI: −0.71 to 5.00 vs. PLA, *n* = 48: +0.06°, 95% CI: −2.85 to 2.97; *p* > 0.05).

### ROM extension

ROM extension was evaluated at visits one (baseline), four (12 weeks), and seven (24 weeks). Baseline values were comparable between both the study groups (*p* > 0.05). After 24 weeks of supplementation, a slight increase in ROM extension of 2.21° was observed in the undenatured collagen group and a slight increase of 1.27° in the PLA group. Analysis of changes over baseline (*p* < 0.01) and over time (*p* = 0.0061) in knee ROM extension showed a significant increase in the undenatured collagen group. No such change was observed in the PLA group (*p* > 0.05, Fig. 2b, Table 2).

### Safety assessments

No abnormalities were reported for any of the blood biochemistry, hematology, or vital signs (Table 1). Similarly, no study product-related AEs were noted in either the undenatured collagen or the PLA group. The tolerability of undenatured collagen was rated as “well tolerated” by 98% of the study participants. Based on this, it is concluded that supplementation of undenatured collagen was well-tolerated over the 24-week study period.

## Discussion

Joint flexibility is of utmost importance in the daily lives of athletes as well as in active people. It has been reported that the stress on knee joints during physical activity may result in immunologic responses that mirror those seen in arthritic diseases, which can ultimately lead to a decrease in knee joint flexibility.^13^ Similarly, aging has been shown to reduce knee ROM due to wear and tear exerted on the joints from daily use.^14–19^ According to the CDC, an average adult loses 1° of knee flexion and extension ROM for every 10 years of age.^18^ This loss in flexibility with age has been attributable, in part, to decreased activity^20^ and decreased joint mobility.^18^ Therefore, restoring and maintaining knee ROM are critical to keeping joints healthy.

Knee ROM is commonly used as an outcome measure in clinical studies of people with knee OA, rheumatoid arthritis (RA), and in athletes.^21,22^ Steultjens et al. demonstrated that the mean knee ROM in an OA group was 19% lower than in control group.^21^ In another study, McCarthy et al. reported that patients with knee OA had significantly lower knee flexion ROM than matched controls.^23^ Other studies have reported that patients with knee OA demonstrate reduced knee motion during walking compared with healthy controls alongside a reduction in gait velocity.^24–26^ In another study, strong correlations were found between the loss of ROM of the knee and hip joints, and disability in an elderly population.^27–29^

In the current study, a significant improvement in knee flexion was observed with undenatured collagen supplementation. An increase in knee flexion was seen as early as 8 weeks of supplementation and it continued to improve eventually reaching 3.23° at the end of the study. In a recently published pilot study, the authors reported that healthy subjects with ArJD have impaired ROM.^11^ The improvement by 3° in the current study suggests that undenatured collagen supplementation may benefit to improve knee flexion in healthy subjects who are at risk of developing joint ailments down the road.

Subgroup analysis based on gender showed that females reported higher ROM flexion improvement in response to undenatured collagen supplementation than was seen in males. This gender-based difference in efficacy could be possibly attributed to the fact that females with joint disorder exhibit lower ROM,^21^ through which supplementation with undenatured collagen might allow for a greater chance to see better outcomes in such a population.

A separate subanalysis based on age demonstrated that subjects aged 35 years and older experienced a higher increase in active ROM flexion in response to undenatured collagen supplementation than was observed with subjects between 20 and 35 years of age. One possible explanation for these preferential results might be due to the fact that older subjects are expected to have lower ROM to start with compared with younger subjects, and hence, one would expect that older adults could benefit to a higher extent from undenatured collagen supplementation.

A significant increase in knee extension was seen in the undenatured collagen group after 24 weeks of supplementation. This is in agreement with the previous research where supplementation of undenatured collagen for 120 days was shown to improve knee extension ROM in healthy subjects who experienced joint pain upon strenuous exercise.^10^ These same subjects were also able to exercise longer before experiencing joint pain post-120 days of supplementation.^10^ In the present study, an increase in knee extension, in addition to flexion, suggests that undenatured collagen supplementation could improve joint function and mobility to better support everyday activities.

With respect to passive knee flexion, no significant changes were observed between the study groups. This is not surprising as subjects in this study were healthy and hence did not have any overt restriction in their joint mobility/movement. In addition to this, considering that passive knee flexion is reached with help from the investigator, many of the study participants were able to reach passive knee flexion to the maximum extent at the beginning of the study. This could possibly explain the lack of significant change in passive knee flexion between the study groups.

Although it has been also shown in previous studies that OA subjects as well as subjects with activity-related joint discomfort may benefit from a supplementation with undenaturated type II collagen by enhancing joint mobility,^9,10^ the exact mechanism of action is still not fully understood. According to animal and *in vitro* studies, it is assumed that during exercise, some processes that also occur in OA are activated, such as distribution of proinflammatory cytokines.^13,30,31^ Undenatured type II collagen appears to reduce joint inflammation by acting via the gut-associated lymphoid tissue. It seems to stimulate immune cells (T cells) to produce anti-inflammatory cytokines in joints.^31^ This mechanism helps to diminish inflammatory processes and to stimulate cartilage repair, which seems to be a possible mode of action of the study product.

The current study has limitations that the reader should consider when reviewing the results. No biomarker assessment was performed in the current study to investigate the mode of action of undenatured type II collagen on joint health. This should be investigated in further studies.

Furthermore, during the study, there was a global outbreak of severe acute respiratory syndrome coronavirus 2 (SARS-CoV-2), leading to the COVID-19 pandemic. For the major period of 2020, gymnasiums, fitness centers, stores, and other public places were all closed as part of a global lockdown aiming to halt the spread of the virus. Some subjects, especially those who regularly performed weight training and team sports—such as soccer and handball—could not participate in their usual physical activities or training.

However, study investigators advised the subjects to continue with their usual physical activity routines and perform alternative sport types at home or outdoors where possible to maintain consistency. Most subjects followed these recommendations; however, during the winter months in colder climates, outdoor physical activities were often not possible. Therefore, some subjects reported a reduction in the frequency of regular physical activity.

As these changes were only transient, these deviations were rated as minor. Because of the COVID-19 pandemic, some rescheduling of study visits had to be made. Nevertheless, the intake period was not interrupted as subjects were supplied with additional products.

Overall, the findings from this study underline the importance of maintaining healthy ROM, especially in subjects who already show signs of impairment. This study showed promising results for undenatured collagen supplementation as a dietary ingredient with the ability to help improve knee joint ROM flexion and extension.

## Conclusions

In the current study, the effect of undenatured collagen supplementation on knee joint flexibility in subjects with ArJD was investigated. The data support that undenatured collagen UC-II is a food ingredient with the potential to positively affect function of knee joint resulting in an improvement of knee flexion assessed by goniometry, demonstrating the benefit in a population at risk. As the biomarker assessment was not performed in the current study, this should be emphasized in future research to investigate the mode of action of undenatured type II collagen on joint health.

## Supplementary Material

Supplemental data

Supplemental data

Supplemental data

Supplemental data

## Data Availability

The data presented in this study are available on request from the corresponding author. The data are not publicly available due to confidentiality and ethical reasons.
