# Supplementary material for: UC-II Undenatured Type II Collagen for Knee Joint Flexibility: A Multicenter, Randomized, Double-Blind, Placebo-Controlled Clinical Study
Source: J Integr Complement Med. 2022 Jun 7;28(6):540–8. doi: 10.1089/jicm.2021.0365 (PMC9232232; doi:10.1089/jicm.2021.0365)

**Figure S1:** Goniometer Measures of Knee Flexion: a) position 1, b) position 2; and Extension: c) position 1, d) position 2.


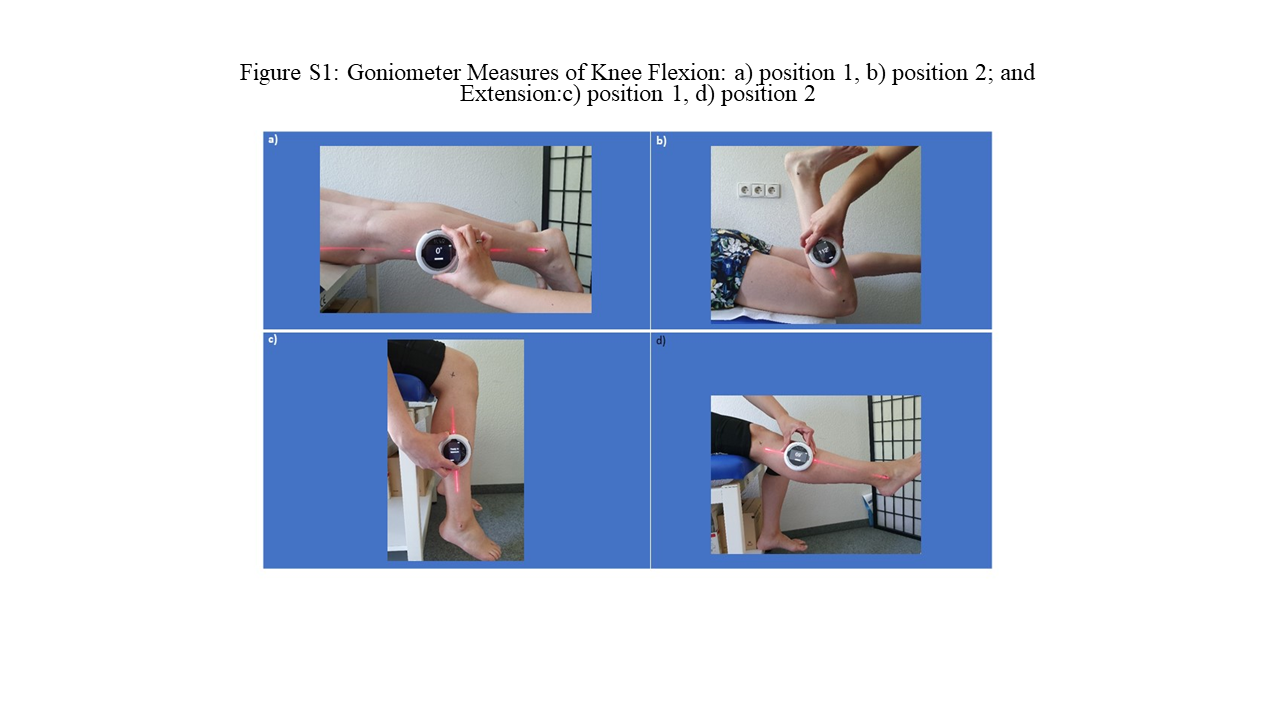

Supplement: Supplemental data [file Suppl_FigureS1.docx]
