# Supplementary material for: UC-II Undenatured Type II Collagen for Knee Joint Flexibility: A Multicenter, Randomized, Double-Blind, Placebo-Controlled Clinical Study
Source: J Integr Complement Med. 2022 Jun 7;28(6):540–8. doi: 10.1089/jicm.2021.0365 (PMC9232232; doi:10.1089/jicm.2021.0365)

**Figure S2**: Change in knee flexion ROM active [°] in Undenatured Collagen group versus PLA group; Scatter diagram with mean ± 95 % CI; *p<0.05. Abbreviations: ROM: range of motion.


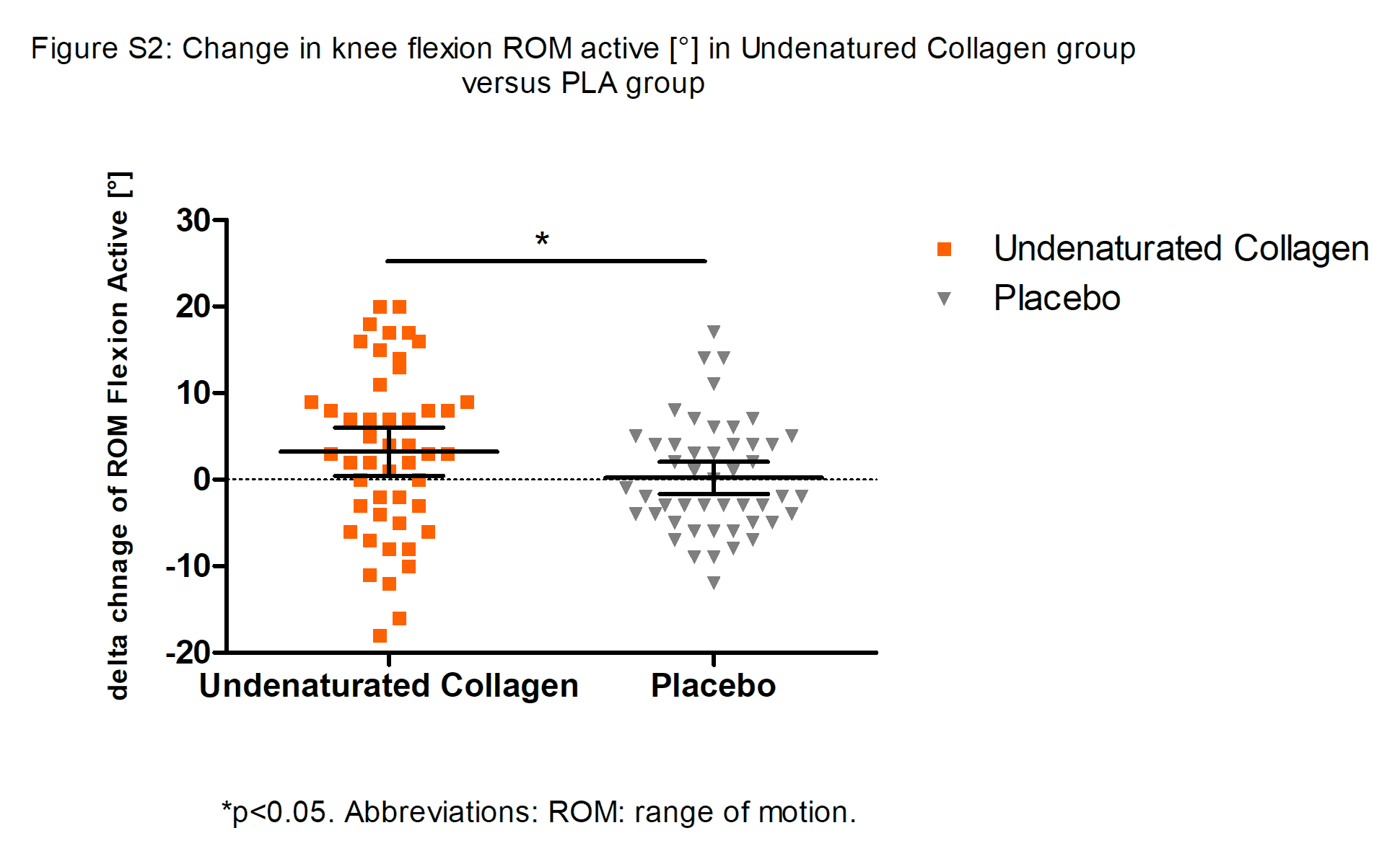

Supplement: Supplemental data [file Suppl_FigureS2.docx]
