# Supplementary material for: UC-II Undenatured Type II Collagen for Knee Joint Flexibility: A Multicenter, Randomized, Double-Blind, Placebo-Controlled Clinical Study
Source: J Integr Complement Med. 2022 Jun 7;28(6):540–8. doi: 10.1089/jicm.2021.0365 (PMC9232232; doi:10.1089/jicm.2021.0365)

**Figure S4**: Distribution of ROM flexion active [°] in subgroup of >35 years old over the study duration; Line graph with mean ± 95 % CI; * p<0.05, ** p <0.01; significant difference between the study groups; †† p<0.01 significance difference over time in Undenatured Collagen group. ROM: range of motion


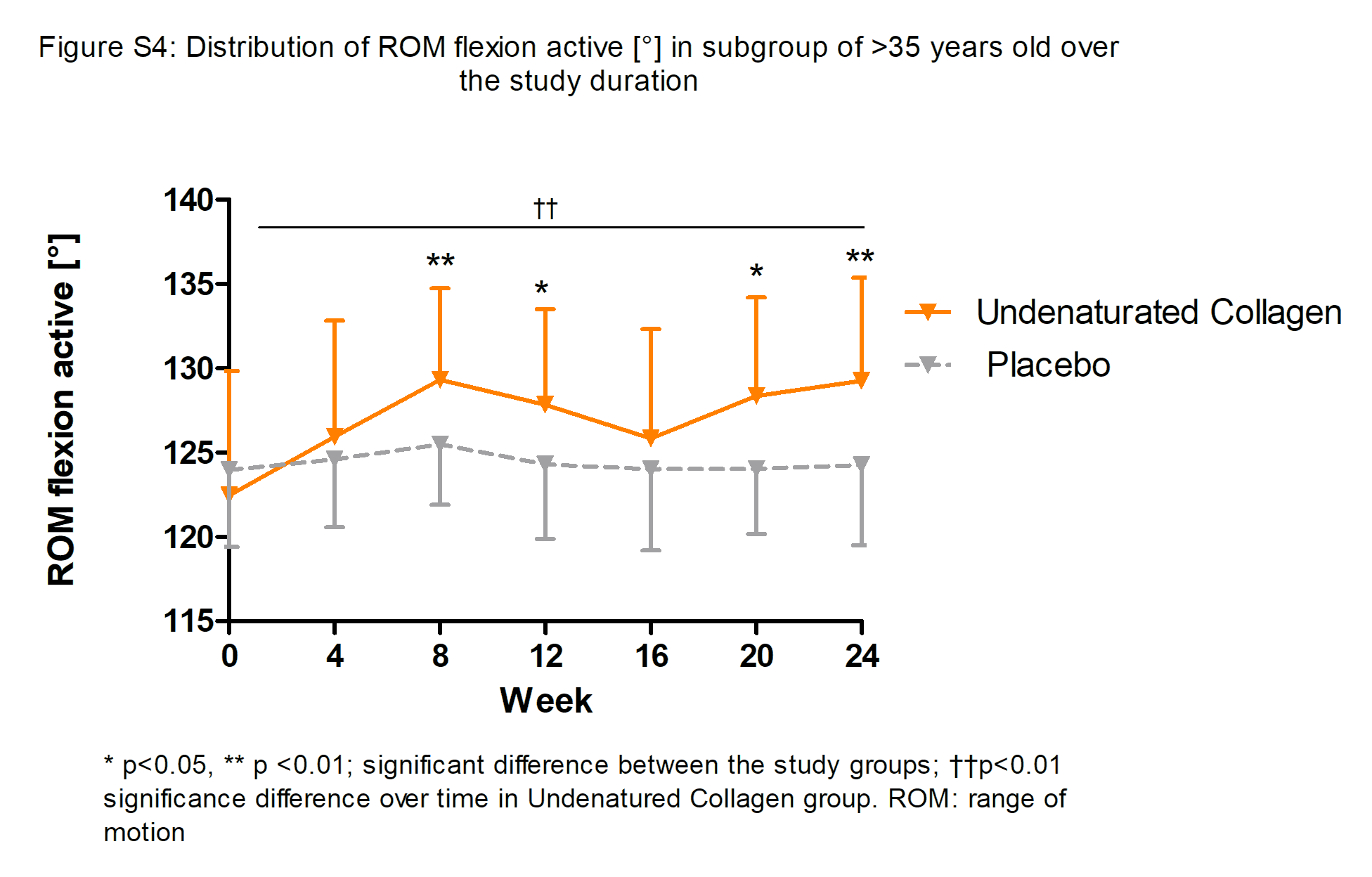

Supplement: Supplemental data [file Suppl_FigureS4.docx]
